# Implementation of a safer conception programme for HIV-affected men and women in rural Uganda

**DOI:** 10.1080/17441692.2024.2342023

**Published:** 2024-05-08

**Authors:** Lynn T. Matthews, Moran Owembabazi, Deogratius Tukwasibwe, Alice Najjuma, Winnie Muyindike, John Mary Tumwine, Benjamin Hornstein, John Bassler, Dustin Long, Elizabeth Gill, Cynthia Young, Pooja Chitneni, Christina Psaros, Micheal Kanyesigye, Paul Kato Kalyebara, Angela Kaida, Mwebesa Bwana

**Affiliations:** a Division of Infectious Diseases, University of Alabama at Birmingham Heersink School of Medicine, Birmingham, AL, USA; b Mbarara University of Science and Technology, Mbarara, Uganda; c Mbarara Regional Referral Hospital, Mbarara, Uganda; d Department of Medicine, Mbarara University of Science and Technology, Mbarara, Uganda; e Department of Biostatistics, University of Alabama at Birmingham School of Public Health, Birmingham, AL, USA; f University of Alabama at Birmingham Heersink School of Medicine, Birmingham, AL, USA; g Division of Infectious Diseases, University of Kentucky, Lexington, KY, USA; h Centre for Global Health, Massachusetts General Hospital, Boston, MA, USA; i Division of General Internal Medicine, Brigham and Women’s Hospital, Boston, MA, USA; j Department of Psychiatry, Massachusetts General Hospital, Boston, MA, USA; k Department of Obstetrics and Gynaecology, Mbarara University of Science and Technology, Mbarara, Uganda; l Faculty of Health Sciences, Simon Fraser University, Burnaby, Canada

**Keywords:** Uganda, HIV, safer conception, treatment as prevention, PrEP

## Abstract

We integrated safer conception care into a Ugandan HIV clinic. People with HIV (PWH), or partnered with a PWH, and desiring children were eligible for the Healthy Families Clinic Program. Clients completed quarterly safer conception counselling visits and questionnaires to provide information around method preferences and outcomes (partner pregnancy, partner seroconversion). We used clinic level data to evaluate longitudinal viral suppression among PWH. Between November 2016 and January 2020, 361 clients (53% men) accessed services. 75% were PWH (51% women, 96% men): 99% were on antiretroviral therapy (ART) and most reported HIV-sero-different partnerships (97%). Frequently selected safer conception methods included ART (86%), timed condomless sex (74%), and PrEP (40%) with important differences by HIV-serostatus and gender. 22.5% reported pregnancy. Most (97%) PWH were virally suppressed at enrolment and 81% of non-virally suppressed PWH were virally suppressed at 15 months. Two HIV-negative clients (2%) had HIV seroconversion. There is demand for safer conception care in a public sector HIV-clinic in Uganda. Men and women have unique safer conception care preferences. The majority of clients engaged in safer conception care had viral suppression at follow up.

## Introduction

In many settings, including Uganda, men and women with HIV or partnered with someone with HIV desire children and may risk HIV transmission in order to meet important reproductive goals (Beyeza-Kashesya et al., 2010; Heys et al., 2009; Kaida et al., 2019; Matovu et al., 2017; Nakayiwa et al., 2006; Pratt et al., 2022; Wagner & Wanyenze, 2013; Wanyenze et al., 2013). Safer conception methods are HIV prevention strategies that allow for conception while minimising HIV transmission and include ART-mediated HIV-RNA suppression, pre-exposure prophylaxis (PrEP), delaying condomless sex until viral suppression is achieved, disclosure of HIV-serostatus, and STI treatment (Davies et al., 2018; Matthews et al., 2012; Matthews et al., 2018; Uganda Ministry of Health, 2020). These methods effectively decrease HIV transmission risk while empowering serodifferent couples to meet reproductive goals (Heffron et al., 2019; Hurley et al., 2022; Schwartz et al., 2019; Wagner et al., 2021; Wagner et al., 2021).

While each of these individual HIV prevention strategies are included within Uganda Ministry of Health’s HIV prevention and treatment guidelines (Uganda Ministry of Health, 2020), patient-centred care and counselling that addresses the reproductive goals of HIV-affected men and women is not routinely integrated into HIV care. Providers in Uganda and elsewhere generally support reproductive rights for serodifferent couples but often lack sufficient training to incorporate these messages into their practice (Gutin et al., 2020; Patwa et al., 2019; Wagner et al., 2021; Young et al., 2021). Stigmas related to HIV and reproductive goals at personal, community, and provider levels towards those impacted by HIV can limit adoption of safer conception strategies and opportunities to engage HIV-affected couples in this care (Gutin et al., 2020; Gutin et al., 2021; Kimemia et al., 2019). Further, studies have shown that while men express interest in safer conception care, they are often excluded from reproductive health conversations (Mathenjwa et al., 2021; Matthews et al., 2021; Short et al., 2021; Stanton et al., 2022). These factors all pose a large gap in care for HIV-affected couples; to address this gap our team launched a pilot safer conception programme in November 2016.

Based on our formative work, pilot programme results (Ashaba et al., 2017; Crankshaw et al., 2012; Greener et al., 2018; Kaida et al., 2019; Kastner et al., 2014; Khidir et al., 2018; Matthews et al., 2016; Matthews et al., 2017; Matthews et al., 2013; Matthews et al., 2015; Matthews et al., 2014; Matthews et al., 2014; Matthews et al., 2016; Matthews et al., 2018; Mosery et al., 2017; Nieves et al., 2015; Young et al., 2018) and using our periconception risk reduction theoretical framework (Crankshaw et al., 2012; Saleem et al., 2017; Ware et al., 2009; Ware et al., 2013; Ware et al., 2012), we integrated safer conception care into an HIV care clinic in rural Uganda. The ‘Healthy Families Program’ is a patient-centred intervention (International AIDS Society, 2022) rooted in cognitive behavioural therapy strategies that supports clients to develop and implement a safer conception plan. Clients can participate as couples or individuals who are HIV-affected (i.e. in an HIV-serodifferent or HIV sero-concordant positive partnership, living with HIV, or HIV-uninfected with unknown-HIV serostatus partnerships). HIV testing and counselling for individuals and couples, support to disclose HIV serostatus to sexual partners, as well as encouragement to engage the desired pregnancy partner in the programme are critical elements. The counselling content addresses internalised stigma around having children while living with HIV and includes messaging that people on treatment with an undetectable viral load cannot transmit HIV to sexual partners (i.e. Undetectable = Untransmittable). The Healthy Families program offers up to three sessions (plus two check-ins) of education and problem-solving, communication skills, and ART adherence skills. We also offer tenofovir disoproxil fumarate/emtricitabine (TDF/FTC) as daily oral PrEP to HIV-uninfected partners with adherence support. All sessions and treatments are provided free-of-charge to clients.

Initiated in November 2016, the pilot programme has provided safer conception care to over 300 HIV-affected individuals and couples who want to have a child. Few studies have described experiences and outcomes of safer conception care delivery in LMIC settings outside of South Africa (Kaggiah et al., 2021; Patwa et al., 2019; Schwartz et al., 2019; Schwartz et al., 2014; Steiner et al., 2016). In this multiple-method paper we describe the characteristics of clients presenting for safer conception care in rural Uganda, the safer conception approaches that they chose to use and why, retention in the programme, and pregnancy and HIV outcomes with data censored in January 2020.

## Methods

### Study site

The Healthy Families pilot project took place in Mbarara, Uganda. Mbarara Municipality (population 195,000) is located in the Mbarara District of Uganda, 275 km southwest of the capital city, Kampala. The Immune Suppression Syndrome (ISS) Clinic, at the Mbarara Regional Referral Hospital, provides care to people living with HIV with a catchment area spanning southwestern Uganda and parts of northern Rwanda. This clinic initiates ART for approximately 1,000 new patients each year.

### Healthy Families program

**Figure 1.**
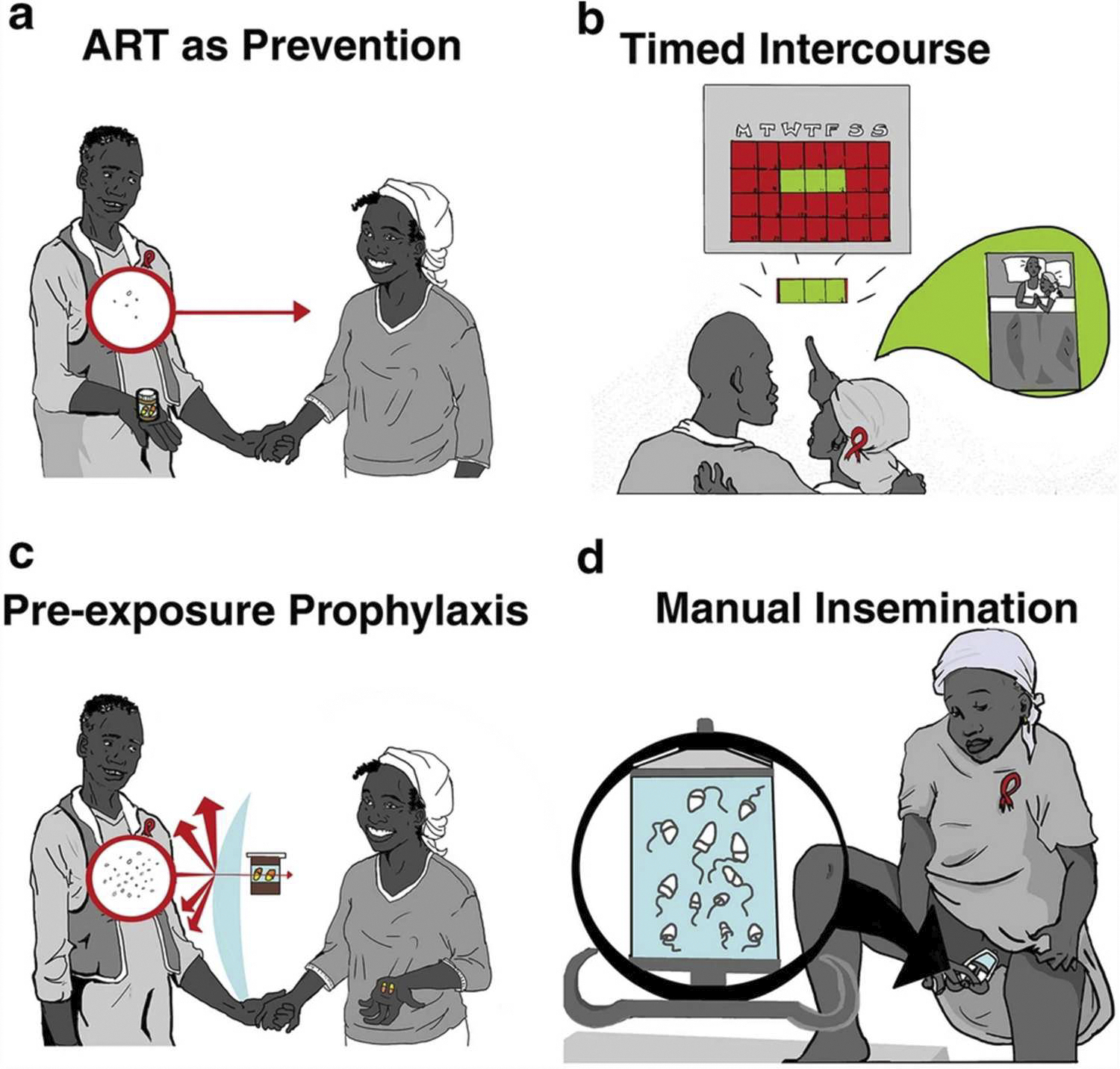
Images used to support education around safer conception methods (Young et al., 2021).

Services began in November 2016 and are available to HIV-affected adults (≥18 years of age) including men and women who express personal or partner desire for pregnancy (this clinic screens for reproductive goals for women and began introducing screening for men with advent of this programme). The counselling programme includes three counselling sessions for clients with or without their pregnancy partners. We use culturally appropriate safer conception counselling content based on prior formative work (Psaros et al., 2014; Psaros et al., 2015), and adapted to the local context with input from safer conception experts and Ugandan HIV healthcare providers. At the time of programme initiation, TDF/3TC or TDF/FTC as PrEP were available in the clinic as an early rollout site for Uganda. Safer conception strategies discussed in the counselling sessions include ART-mediated viral load suppression, couples-based HIV counselling and testing, PrEP for HIV-exposed uninfected partners, condomless sex timed to peak fertility, STI treatment, and self-insemination for couples in which the woman is living with HIV. We also discuss semen processing for couples in which the man is living with HIV, services which are available in the capital city, however, largely inaccessible to most HIV-affected couples in Uganda (Figure 1). During the counselling sessions, we assess HIV disclosure and pregnancy goals, offer HIV testing to verify HIV serostatus of clients and partners, measure HIV-RNA viral load for participants living with HIV, and conduct syndromic screening and treatment for STIs. Pregnancy partner participation is strongly encouraged, but not required.

Healthy Families program clients were invited to participate in related studies including a study evaluating PrEP uptake and adherence for HIV-uninfected women planning to have a child (Matthews et al., 2023) and a study evaluating outcomes of safer conception care on HIV-RNA and retention in care for men living with HIV (Chitneni et al., 2020).

### Counsellor training and support

The Healthy Families program is led by a clinic counsellor (DT), who is trained as a midwife and HIV counsellor, and a nurse (AN), who is an experienced HIV clinical care provider. Two physicians with expertise in HIV and safer conception care (MB and LTM), provided initial didactic training in safer conception content and methods and provided ongoing onsite support and booster trainings. The counselling content includes elements of education, problem solving, and PrEP adherence support, and described in detail elsewhere (Khidir et al., 2018). The clinic counsellor and the nurse were trained in these methods (by CP) over Skype, then during an in-person collaborative training session in South Africa (Matthews et al., 2022), and supported with a monthly counselling training and support call for the Uganda Healthy Families clinic and the South Africa research team.

The Healthy Families nurse and counsellor led weekly team discussions about challenging cases with Ugandan and U.S. providers who had expertise in HIV care as well as obstetrics and gynaecology.

### Data collection and analysis

#### Quantitative data

At enrolment and 3-monthly follow-up visits, the Healthy Families program counsellor collected clinical data on clients and partners (if present) using paper charts. Data captured included individual characteristics (age, HIV serostatus, ART use, months trying for pregnancy), partnership characteristics (marital status, number of children with current partner, partnership HIV serostatus, whether or not the pregnancy partner attended or was willing to attend the programme, disclosure of HIV status and reproductive goals to pregnancy partner).

At enrolment and 3-monthly follow-up visits, all clients and partners with unknown or HIV-negative serostatus were offered HIV testing, conducted as per national Ugandan Ministry of Health (MOH) HIV testing guidelines, including rapid fourth generation testing for HIV1/2 as screening (Uganda Ministry of Health, 2020). Those living with HIV were offered HIV-RNA testing conducted as per Uganda MOH guidelines offered by the clinic, 6-monthly in the first year of ART and annually thereafter. Female clients and partners were offered pregnancy testing via urine b-hcg testing. All clients and partners underwent syndromic STI management and treatment (Uganda Ministry of Health, 2020).

All data from paper charts and testing results were entered into the online medical record system as part of routine clinic care (International Epidemiology Databases to Evaluate AIDS (IeDEA), 2022) and abstracted for this analysis.

#### Quantitative data analysis

Analyses began by summarising available data using measures of central tendency (sample medians), dispersion (interquartile range), and distribution (frequency, percentage). Hypothesis testing (Kruskal–Wallis, Pearson Chi-Square test; Mantel–Haenszel Row Mean Scores test) was utilised when appropriate statistical assumptions and sample sizes were met and evaluated at the 0.05 significance level. Missing data was considered missing at random. No imputation methods were used and missing data were not included in descriptive measures or statistical tests.

Safer conception method preferences of index clients were assessed using frequencies, stratified by sex and index client HIV status. Programme retention and HIV outcomes were examined among index clients with at least 90 days of follow-up from enrolment and were assessed overall and stratified by index client sex and HIV status. Baseline viral load status was assessed using the closest available clinic viral load data to the participant’s enrolment date (either up to 455 days before or up to 40 days after, whichever is closest to enrolment) based on chart review (median 78 days prior to enrolment). Subsequent viral load status considers all available testing that occurs at least 40 days after enrolment and prior to the data capture end date. The Kaplan–Meier Method was used to describe time to viral suppression, based on viral suppression status at baseline. Pregnancy incidence and outcomes (livebirth, miscarriage, other outcomes) were assessed based on pregnancy tests, participant and participant partner self-report. All analyses were conducted using SAS software, version 9.4 of the SAS System for Windows.

#### Qualitative data

In-depth individual interviews were conducted with providers, index clients, and a subset of partners who enrolled in an implementation evaluation study from April through December 2017 (Young et al., 2021). Eligibility requirements included that index clients, partners and providers spoke either English or the local language, Runyankole, and were able to consent. In addition, index clients must have attended at least one counselling session. Partners must have been partnered to index clients who attended at least one session and knew their partner’s HIV serostatus. Providers were recruited from clinical settings that were likely interfacing with clients impacted by HIV and with reproductive desires. Eligibility required they were a physician, nurse, peer educator or midwife within the HIV clinic or departments that likely worked with clients enrolled in the programme. Interviews occurred in private clinic locations. They were conducted in the participant’s preferred language (English or Runyakole) by a trained Ugandan research assistant, fluent in both languages. Interviews lasted approximately an hour and were audio recorded. All audio-recorded interviews were translated to English and transcribed by the interviewer.

Ten field observations were conducted in real-time during counselling sessions within this timeframe. Clients and partners were approached by a research assistant prior to their session, and if clients were both willing and consented, they were observed by the research assistant during their counselling session. The research assistant did not interact but took detail notes during these sessions (Young et al., 2021).

#### Qualitative data analysis

Researchers read and discussed all transcriptions of interviews and counselling observations to create a coding schematic for both client and partner. All transcriptions were coded using NVivo 10 and 12 for analysis. Coding was compared between two researchers for reliability (kappa = 0.85 and 0.98 for client/partner and provider interviews respectively, both indicative of ‘strong’ agreement) (Young et al., 2021). The research team used a thematic analysis approach to develop a primary narrative (Elo & Kyngäs, 2008; Hsieh & Shannon, 2005; Sundler et al., 2019). This paper reports data identified through a descriptive code regarding safer conception method preferences. The team’s previous work published on barriers and opportunities for the safer conception care programme to support couples to safely meet reproductive goals but did not focus on method preferences (Young et al., 2021).

### Ethical considerations

This quantitative data collection study was approved as a chart review by Mbarara University of Science and Technology and the institutional review boards at Partners Healthcare (Boston) and at University of Alabama at Birmingham. Data were available based on the clinic serving in the IeDEA Consortium (MNRH ISS CLINIC Database) and the IeDEA Consortium (National Institutes of Health U01 AI069911). In-depth interviews were collected as part of a research study approved by Mbarara University of Science and Technology-Research Ethics Committee (MUST-REC) and the institutional review boards at Partners Healthcare (Boston) and at University of Alabama at Birmingham.

## Results

### Characteristics of index clients at enrolment in the Healthy Families program

Between 7 November 2016 and 27 January 2020, 361 index clients including 170 (47%) women and 191 (53%) men enrolled into the Healthy Families program. Enrolled women were younger (median = 28.6 (IQR 25.3, 33.7)) than men 36.7 (IQR 31.6, 43.9) (*p* < 0.001). Almost all (*N* = 351, 98.0%) clients reporting being married (or living as married) to their desired pregnancy partner and 94% (*N* = 226) had one or more children with their desired pregnancy partner.

Most men index clients (*N* = 183 (96%)) were living with HIV (MWH). About half (*N* = 86, 51%) of women clients were living with HIV (WWH). At programme entry, virtually all index clients with HIV reported accessing ART (*n* = 268; 99.6%). The majority of participants (*N* = 78, 92% of women and *N* = 158, 91% of men) with HIV were virally suppressed (HIV-RNA ≤ 550 copies/mL). Most PWH (*N* = 77, 94% of women and *N* = 165, 98% of men) reported disclosure of HIV-serostatus to their desired pregnancy partner. Of those who reported their partner’s HIV status, 98% (*n* = 126) of women and 99% (*n* = 186) of men reported an HIV-uninfected or unknown serostatus partner.

At time of enrolment in the programme, women clients reported trying for pregnancy for a median of 6 months (IQR 2.5–12.0) prior to accessing the programme. Most clients (96% of both women and men) reported having previously discussed their reproductive goals with their desired pregnancy partner. Among women, 16% attended the first counselling session with their desired pregnancy partner, compared with 27% of men (*p* = 0.015). However, 81% of women and 95% of men reported that their desired pregnancy partner would be willing to attend the programme with them (Table 1).

### Safer conception method selection at enrolment

Across 335 index clients with data on choice of safer conception method (93% of total enrolled), most (88%) selected ART with HIV-RNA suppression as their preferred method for safer conception. Timed condomless sex (selected by 75%), and PrEP for uninfected partners (selected by 40%) were also frequently selected. Three-quarters of clients (75%) chose to use more than one safer conception method.

**Figure 2.**
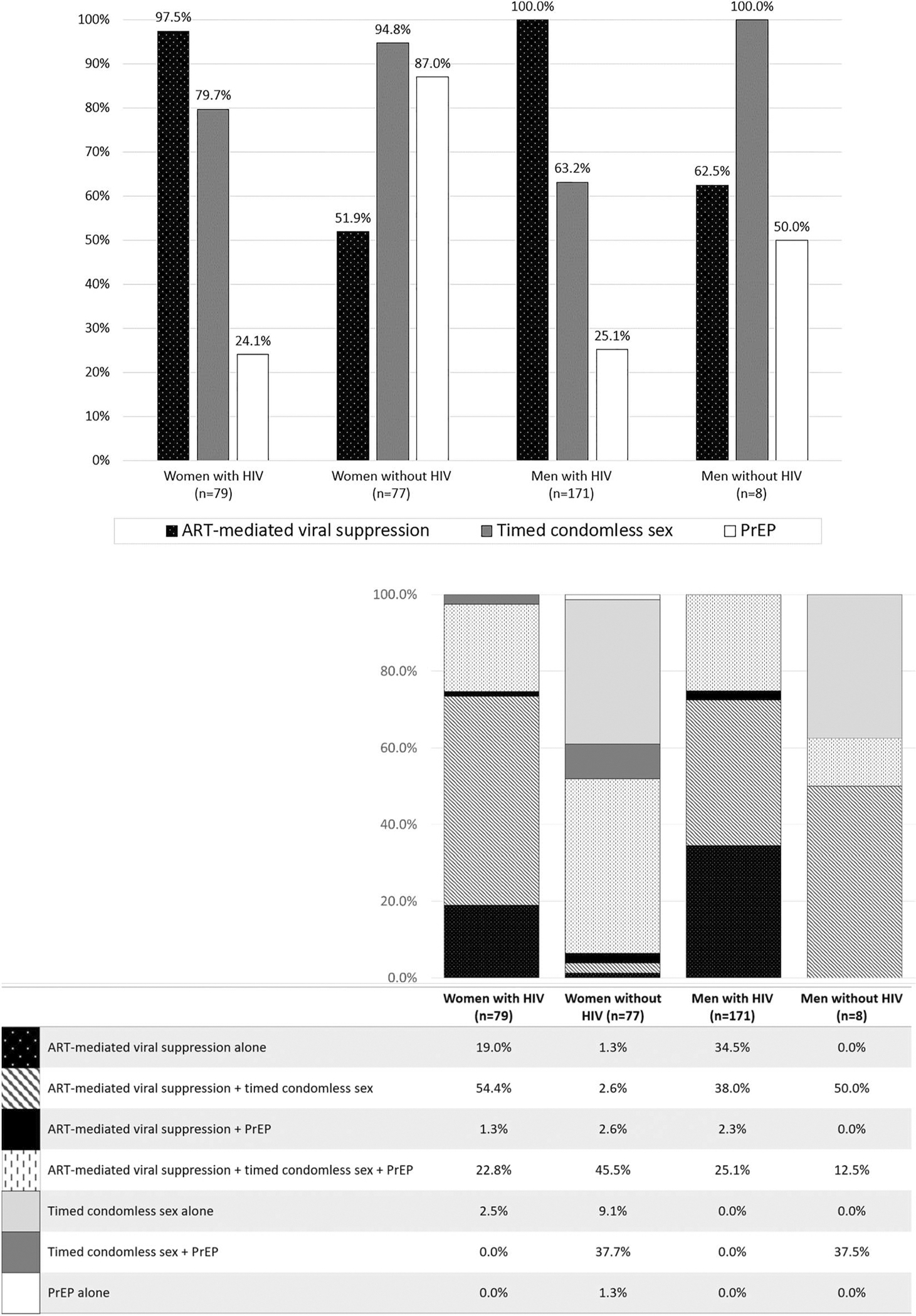
Safer conception method preferences of index clients of the Healthy Families Clinic program after first counselling session by gender and HIV status. (a) Preferences for the top three safer conception methods. (b) Preferences for multiple and overlapping safer conception methods.

We observed differences in safer conception method choice by gender and HIV-serostatus. Among MWH (*N* = 174), all chose ART-mediated viral load suppression. 63% also chose timed condomless sex, while 27% also chose PrEP for their partner. Among WWH (*N* = 80), almost all (98%) chose ART-mediated viral load suppression. Seventy-seven percent also chose timed condomless sex, and 23% selected PrEP for their partner. Among HIV-uninfected women (*N* = 77), 95% chose timed condomless sex, 87% chose PrEP, and 52% chose ART-mediated viral suppression for their HIV-positive partner (Figure 2(a) and (b)).

### Qualitative findings on safer conception method preferences

Qualitative data similarly revealed that clients preferred to use ART-mediated viral suppression and timed condomless sex, but expressed uncertainty about how safe these methods were in practical usage. For instance, this MWH with viral suppression expressed his worries with relying solely on ART-mediated viral load suppression to prevent HIV transmission to his HIV-negative partner alongside the challenges of timing condomless sex to peak fertility:

We discussed with the counsellor … he wrote for us on a paper how her menstrual circle goes. He told us the fertile days and when we reached at home, we kept on discussing it. But we found out that we may not manage to follow that, so we decided that we can target after her periods then I can sleep with her without a condom. The counsellor said if I am taking my drugs well, we can even spend a whole month having unprotected sex, but for me I fear to do that I can only do it after her periods like for 3 days and I stop.

-Man with HIV, Interview #13 (virally suppressed)

Concerns and uncertainty about the efficacy of ART-mediated viral suppression for HIV prevention led some participants to add PrEP use (for themselves or their HIV-negative partner), to provide additional protection during conception attempts. This MWH describes how he and his partner spoke with the counsellor about their options:

They also explained to her that I had tested my viral load and they told her that it was suppressed. The counsellor told her that medically I would not be infecting her because my viral load is already suppressed […] According to other studies they say I cannot infect any one with the virus because of my viral suppression so even if she does not take PrEP nothing will happen, but she refused and opted for PrEP.

-Man living with HIV, Interview #5 (virally suppressed)

The following HIV-uninfected women described her motivations for periconception PrEP as a need to protect herself and not depend on her partner’s pill-taking behaviour.

I wanted to protect my life so that I do not get infected with the virus because my husband may fail to swallow his drugs. He may be away from home, and you cannot know if he has swallowed or not since you have not gone with him. When he comes back, he wants you to have sex with him. But when I am using this drug, whether he swallows or not, this drug will help me.

-Woman without HIV, Interview #75

Other safer conception methods were less frequently discussed. For instance, we observed low uptake of self-insemination or sperm washing driven by cultural preferences as well as access difficulties in the region. As this provider describes:

The things that we talk about like sperm washing and insemination are not very well accepted in the community, and they are expensive. We offer sperm washing in this centre, but it goes between 2.5 million to 3 million shilling [~700–850 USD] and when you compare this to a couple just sleeping together for a few minutes and they get a child at zero cost, it makes a very big difference. So, I still insist that viral load suppression will go a long way to help us.

-Female, OBGYN Doctor, Interview #34

Methods promoting manual home insemination were infrequently selected. In the following counselling observation, the observer documents barriers to uptake of home insemination for safer conception.

When he [clinic counsellor] talked about the self-insemination, the man [client] laughed and asked the counsellor if he has had any cases of people who like that. The counsellor said that there are. Then the man, while looking down, said that for him he cannot allow his wife to do such a thing because it is not accepted in the society. And that even he thinks even if the child is conceived by that, that child can have defects. The counsellor told him that that is not true, but he seemed not to be convinced because he looked away as he told him that.

-Observation #6

### Programme retention: Index client follow-up visits

**Figure 3.**
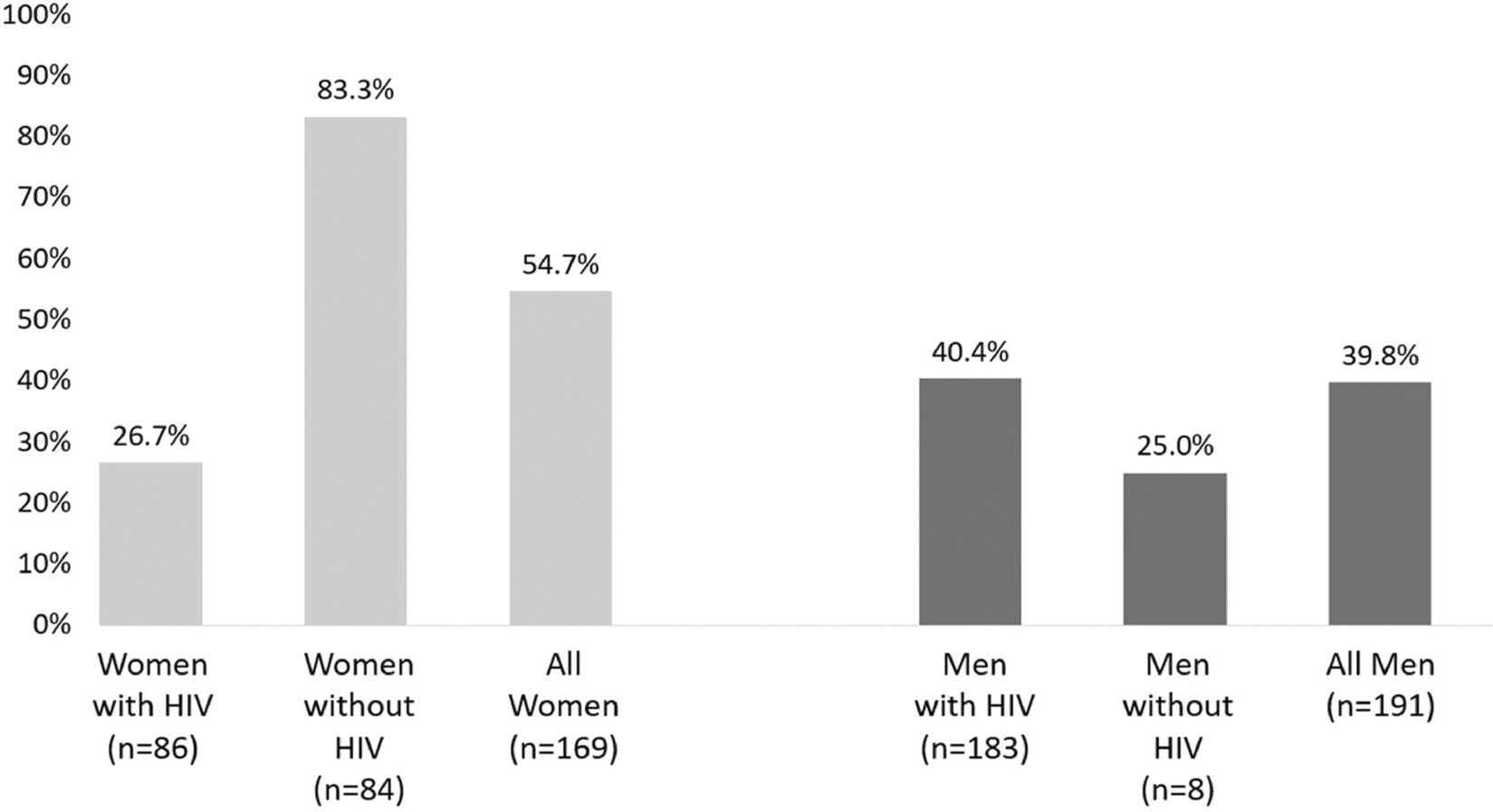
Proportion of index clients enrolled in the Healthy Families Clinic program who returned for at least one follow-up visit, by gender and HIV status (*n* = 360).

Among 360 index clients (169 women, 191 men) who were eligible for a follow-up visit (i.e. at least 90 days since enrolment by cohort censor date; 99.7% of the enrolled cohort), 93 (55%) of women and 76 (40%) of men returned for at least one follow-up counselling visit. Of those who returned, median months between enrolment and the first follow-up visit was 3.1 [IQR: 2.0, 3.6] for women and 2.9 [IQR: 1.9, 3.2] for men. A lower proportion of WWH than WWoH returned for a follow-up visit (26.7% vs 83.3%; *p* < 0.0001 by Pearson Chi Square) while a higher proportion of MWH than MWoH returned for a follow-up visit (40.4% vs 25.0%; *p* = 0.4808, by Fishers Exact Test) (Figure 3).

Among index clients who attended a follow-up visit, 13% (12/93) of women attended with their desired pregnancy partner compared with 63% (48/76) of men. Clients living with HIV (WWH (26%) and MWH (64%)) were more likely to attend their follow-up visits with their desired pregnancy partner relative to clients without HIV (WWoH (8.7%) and MWoH (50.0%)), although numbers among MWoH were small.

### Pregnancy and HIV outcomes

Among all index clients enrolled in HF program (*n* = 360), 81 (22.5%) reported a pregnancy or partner pregnancy over the follow-up period, including 33 (20%) women and 48 (25%) men. WWH were less likely to experience a pregnancy compared with WWoH (8.1% vs 31.0%; Mantel-Haenszel test statistic = 11.78; *p* = 0.0006). Among men, MWH and MWoH were equally likely to report a partner pregnancy (25.1% vs 25.0%).

Of 81 clients who reported a pregnancy, the most recent pregnancy outcome included: 63.0% were still pregnant at follow-up, 25.9% had a livebirth, and 11.1% experienced a miscarriage or other pregnancy outcome. Among women clients who reported a pregnancy (*n* = 33), 69.7% were still pregnant at follow-up, 15.2% had a livebirth, and 15.2% has a miscarriage of other pregnancy outcome, with little difference by HIV status. Among men who reported a partner pregnancy, 58% of the partners were still pregnant at follow-up, 33% had a livebirth, and 8% experienced a miscarriage or other pregnancy outcome.

### Viral suppression

**Figure 4.**
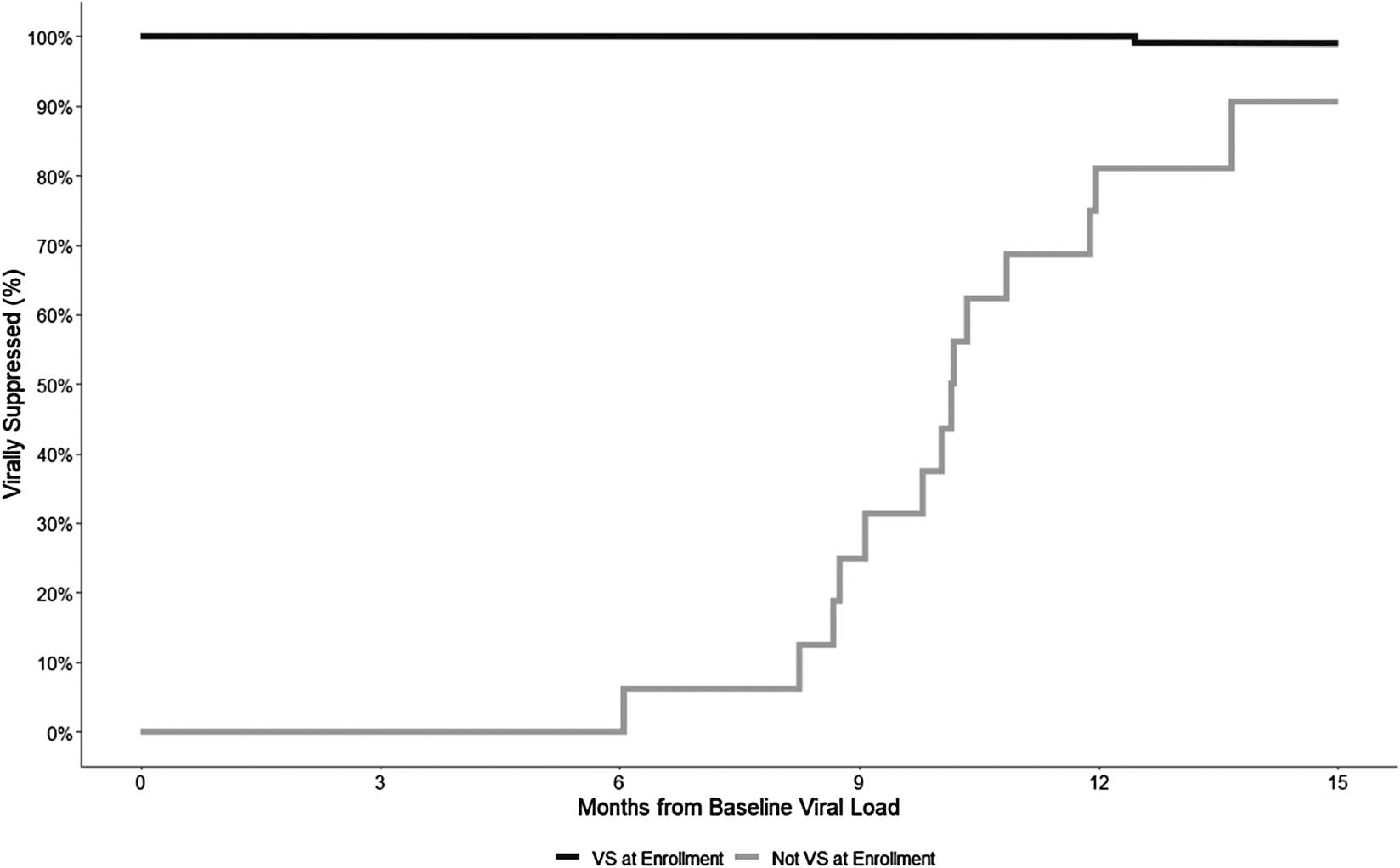
Proportion of clients HIV-virally suppressed over 15 months post-enrolment separated by those with (*N* = 165) and without (*N* = 16) HIV-viral suppression (VS) at enrolment.

Among 269 index clients living with HIV at enrolment, 205 (90.3%) were virally suppressed. At 12 months, among those with follow-up data, 100% (162/162) were virally suppressed; at 15 months, 97% (162/165) remained virally suppressed (Figure 4).

Among 16 clients who were not virally suppressed at enrolment and had at least one additional HIV-RNA test over the follow-up period, 14 (81%) became virally suppressed within 15 months of follow-up (Figure 4). All four (100%) WWH who were unsuppressed at enrolment became suppressed, compared with *N* = 10 (83%) of MWH.

Among 92 enrolled HIV-uninfected index clients, 82 had follow-up testing and two sero-converted (2% of enrolled HIV-uninfected index clients). Both were women.

## Discussion

These data demonstrate demand for and feasibility of an integrated safer conception programme in a rural Ugandan setting that includes a diversity of clients: men and women, individuals and couples, and those living with and without HIV. Over half of index clients were men, a vast majority of whom (96%) were living with HIV. Comparatively, over half of women accessing services were HIV-uninfected. Men were more likely to engage their partner in safer conception care than women (28% vs 12%). Safer conception strategy choice was largely based on ARVs – ART for those living with HIV and PrEP for those without HIV – with most clients selecting multiple methods. Retention in care was high for HIV-uninfected women. At 12 months, 100% of women and 60% of men were virally suppressed, although reporting was incomplete. Approximately 25% of index clients reported a personal or partner pregnancy during follow-up with two seroconversions observed among HIV-uninfected clients.

These findings are consistent with those reported from South Africa, home of the first clinical safer conception programme implemented within an HIV specialty clinic in an HIV-endemic setting (Davies, 2019; Schwartz et al., 2019). This programme also demonstrated high acceptability and value of information provided, and among clients with HIV, high ART uptake and viral suppression (Davies et al., 2019). A related safer conception programme integrated into a primary health clinic in Johannesburg further demonstrated the potential for such care to reduce HIV incidence (Schwartz et al., 2019). Our findings also build on safer conception research in other sites in Uganda, which has emphasised opportunities for reducing periconception transmission among women living with HIV (Wagner et al., 2021). Leveraging this prior work, we demonstrated feasibility in a rural Ugandan setting and show the potential for safer conception programming to serve both men and women, as well as clients living with or without HIV. Indeed, safer conception clients without HIV were more likely to return for follow-up visits compared to clients with HIV. These findings suggest that those without HIV may require more time/visits to benefit from safer conception care, given likely less knowledge regarding HIV transmission. Whereas for people with HIV and engaged in HIV care, safer conception education may be provided in fewer sessions and integrated into routine HIV care.

While much safer conception care is directed towards women, we observed that integrating HIV and reproductive health care engages men. Because gender norms often position men to engage their partners, reaching men provides an opportunity to link their partners to testing, ART, or PrEP as indicated. Our programme successfully engaged men who have sex with women, a population that is difficult to involve in care and treatment (Adeyeye et al., 2018). Our programme leadership included a male counsellor and was championed by a male HIV physician. In addition, our counselling and educational tools were specifically developed to include men (Khidir et al., 2018) based on a successful safer conception pilot intervention for men with HIV in urban South Africa (Matthews et al., 2022). Over half of index clients were men, 28% engaged their partners in care, 40% returned for follow-up, and 98% of those living with HIV were/remained virally suppressed at 12 months. Future work is needed to reach men who are not already established in HIV care.

Women accessing safer conception care were younger, more likely to be HIV-uninfected, and more likely to have an unknown serostatus partner compared to men. The majority of women without HIV desired PrEP as a safer conception method, suggesting that women do not want to depend on their partner’s HIV viral suppression for their own protection. While PrEP may not be cost-effective for safer conception when their partner living with HIV achieves viral suppression (Hoffman et al., 2015), we highlight the importance of periconception PrEP for women who lack certainty regarding partner viral suppression. PrEP was also selected by partners who feared HIV transmission even when their pregnancy partner had an undetected viral load. Conversely, our qualitative findings also document fear and stigma associated with PrEP use among HIV-uninfected partners (Atukunda et al., 2022). The complex implications for HIV-uninfected partners taking PrEP have been documented (Ngure et al., 2016; Patel et al., 2016). Framing education around U = U may assist participants to better understand PrEP risks and benefits. While adherence to periconception PrEP has been high (Matthews et al., 2023) in some settings, including in a sub-study we conducted in the same clinic (Atukunda et al., 2022; Matthews et al., 2023), in others it has been more variable (Matthews et al., 2022). Further research on how to support effective periconception PrEP use is needed.

While we did not require partner participation, about 20% of index clients involved their partner in at least one visit. Women were less likely than men to attend visits with their partner. For future programmes, it is important to consider gender when considering a couples-based vs individual approach to optimise reach.

We observed (and explored in our qualitative research (Young et al., 2021)) that many clients took a multi-layered approach embracing multiple safer conceptions strategies. Given that most clients with HIV enrolled with viral suppression, these data highlight the need to promote U = U messaging. In communities where frequent HIV and viral load testing are uncommon and as providers become more knowledgeable and forthcoming about U = U science (Bor et al., 2021), discussing multiple HIV prevention strategies may optimise client confidence in their safer conception plan. Our work emphasises the importance of choice and offering multiple strategies given method preference differences by gender and HIV-serostatus (Pollock et al., 2017).

Across all gender and HIV status groups, timed condomless sex was a preferred method either alone or in combination with ARV-based methods. Qualitative findings revealed, however, that couples find timed condomless sex difficult to use in practice. These findings raise implications for safer conception guidelines. Firstly, despite its preference popularity in our study and elsewhere (Hancuch et al., 2018; Ngure et al., 2017; Schwartz et al., 2017), when used alongside ART or PrEP, timed condomless sex offers little to no additional HIV prevention benefit. Moreover, participants struggled to understand the fertile period of a woman’s menstrual cycle, limiting the effectiveness of timed condomless sex as a strategy to increase the likelihood of conceiving. Others have shown that providers have difficulty explaining timed condomless sex to peak fertility (Ngure et al., 2017) and women have difficulty accurately determining their day of ovulation (Pfeifer et al., 2017; Zinaman et al., 2012). A recent Cochrane review found insufficient data to determine if timing intercourse to peak fertility increased pregnancy rates (Manders et al., 2015). Collectively, these findings suggest that in the context of access to ARVs for people living with or affected by HIV, training and promotion of timed condomless sex is worth re-examining as a safer conception method.

No clients chose to access semen processing or self-insemination services. These strategies are not aligned with HIV prevention outside of conception, and we maintain that routinely counselling on these methods may not be efficient given that it is inaccessible to the majority of clients seeking safer conception care and many clients express concerns about these methods (Finocchario-Kessler et al., 2014; Mantell et al., 2017; Mindry et al., 2016; Schwartz et al., 2017; Schwartz et al., 2016; Taylor et al., 2013).

Pregnancy incidence data are limited given low follow-up and lack of objective reporting data. Pregnancy was less common among women with HIV (as seen in programmes with more complete follow-up (Schwartz et al., 2019)), however this may reflect more limited follow-up among women with HIV. At enrolment, index women reported trying for pregnancy for a median 6 months (IQR 2.5, 12) prior to accessing the programme. These data were not collected for men, but men who experience infertility may also seek care to support childbearing (Barden-O’Fallon, 2005; Dyer et al., 2004). Thus, this programme may attract people with fertility support needs. Given that undiagnosed infertility is common and may contribute to HIV-exposure (Pratt et al., 2022), programmes offering safer conception care should explore integration of infertility screening and support, including STI care (Chitneni et al., 2020).

This study has limitations. Without a control group, we have limited opportunity to make conclusions about the impact of safer conception programming on outcomes. In addition, some clients accessing care through the clinical programme were also participants in research programmes evaluating PrEP use for women planning for pregnancy (Matthews et al., 2023) or safer conception care for men (Chitneni et al., 2021) which may have improved follow-up. Data for this cohort were obtained through a clinic pilot programme with clinical data abstracted from a public sector clinic: records for HIV testing, partner data, pregnancy testing, and infant outcomes are thus incomplete. The clinical data included variables with missing values. Missing data was assessed to be missing at random and multiple imputation was not pursued. It is possible that estimates are biased, particularly for those variables with >5% missing data, including viral load suppression (Jakobsen et al., 2017). We do not believe that likelihood of missingness was associated with viral suppression. However, if all those with missing viral load data were truly not suppressed, then the proportion of unsuppressed would increase from 9.7% to 23.5%. However, there would still be no difference in suppression by gender. Finally, because the programme did not actively recruit clients, our cohort likely includes people with unmet reproductive goals and/or those with particular interest in HIV prevention in the context of conception. However, clinical data from a public sector pilot programme may provide more generalisable insights into safer conception programming uptake and outcomes.

## Conclusions

This study highlights the opportunity safer conception care has to support people with HIV to remain in care, suppress HIV virus, and ultimately reduce HIV transmission. Further we observed high PrEP uptake in this context. Integrated safer conception care programmes can thus support the treatment and prevention of HIV and goals of eliminating mother-to-child transmission. While there are important benefits, more work is needed to continue reaching individuals most at need, particularly partners with HIV who are not diagnosed or accessing care. This study reflects that a reduced number of visits may be preferred, particularly for those living with HIV, who could best access this information directly integrated with their HIV care. Those desiring a pregnancy with a partner living with HIV may benefit from more comprehensive programming that educates clients on treatment as prevention, while integrating PrEP and other prevention options into their care. Future work to evaluate outcomes and costs of efficient safer conception care into clinics is needed to support implementation.

## Figures and Tables

**Table 1.** Enrolment characteristics of index clients of the Healthy Family program, November 7, 2016-January 27, 2020 (*n* = 361).

	Gender			
	Women (*N* = 170)	Men (*N* = 191)	Total (*N* = 361)	*P*-value

Age (years)	28.6 (25.3, 33.7)	36.7 (31.6, 43.9)	32.7 (27.5, 38.9)	<0.0001^1^
HIV-serostatus, *n* (%)				<0.0001^2^
HIV-positive	86 (50.6%)	183 (95.8%)	269 (74.5%)	
HIV-negative	84 (49.4%)	8 (4.2%)	92 (25.5%)	
Clients with HIV accessing ART (*N* = 269*)	86 (100.0%)	182 (99.5%)	268 (99.6%)	0.4922^2^
Clients with HIV-seroconversion	2 (2.4%)	0 (0.0%)	2 (2.2%)	0.9999^3^
Viral load suppression (HIV-RNA ≤ 550 copies/mL) among clients living with HIV				
Suppressed	68 (93.2%)	137 (89.0%)	205 (90.3%)	0.3190^2^
Not suppressed	5 (6.8%)	17 (11.0%)	22 (9.7%)	
Missing	12	29	41	
Months trying for a pregnancy	6.0 (2.5, 12.0)	-	-	-
Married or living as married w/ pregnancy partner				0.1068^2^
Single	5 (3.0%)	1 (0.5%)	6 (1.7%)	
Married	161 (96.4%)	190 (99.5%)	351 (98.0%)	
Widowed	1 (0.6%)	0 (0.0%)	1 (0.3%)	
Missing	3	0	3	
Children with pregnancy partner				0.3487^4^
No children	9 (8.8%)	5 (3.6%)	14 (5.8%)	
One child	34 (33.3%)	47 (34.1%)	81 (33.8%)	
Two children	36 (35.3%)	48 (34.8%)	84 (35.0%)	
Three or more	23 (22.5%)	38 (27.5%)	61 (25.4%)	
Missing	68	53	121	
Partnership serostatus				0.2238^2^
HIV-seroconcordant (+)	1 (0.8%)	1 (0.5%)	2 (0.6%)	
HIV-seroconcordant (−)	2 (1.6%)	0 (0.0%)	2 (0.6%)	
HIV-serodifferent	126 (97.7%)	186 (99.5%)	312 (98.7%)	
Missing	41	4	45	
Pregnancy partner attended first counselling session	27 (16.2%)	51 (26.8%)	78 (21.8%)	0.0149^2^
Missing	3	1	4	
Disclosed serostatus to pregnancy partner, by client HIV status				
Yes, HIV+	77 (93.9%)	165 (98.2%)	242 (96.8%)	0.0690^2^
No, HIV+	5 (6.1%)	3 (1.8%)	8 (3.2%)	
Missing	4	15	19	
Yes, HIV−	47 (58.8%)	7 (100.0%)	54 (62.1%)	0.0310^2^
No, HIV−	33 (41.3%)	0 (0.0%)	33 (37.9%)	
Missing	4	1	5	
Discussed reproductive goals with pregnancy partner	163 (95.9%)	182 (96.3%)	345 (96.1%)	0.8397^2^
Missing	0	2	2	
Pregnancy partner willing to attend programme				0.0004^2^
Yes	138 (81.2%)	179 (94.7%)	317 (88.3%)	
No	6 (3.5%)	2 (1.1%)	8 (2.2%)	
Do not know	26 (15.3%)	8 (4.2%)	34 (9.5%)	
Missing	0	2	2	

Table statistics reported as median (IQR) for continuous factors, and frequency (column percentage %) for categorical factors. Missing data is reported and not included in summary statistics.

* Limited to *N* = 269 clients living with HIV.

1 Kruskal—Wallis *p*-value;

2 Pearson Chi-square *p*-value;

3 Fisher exact *p*-value;

4 Mantel-Haenszel row mean scores *p*-value.

## Data Availability

All data used in this analysis is available in the Harvard Dataverse Repository, https://doi.org/10.7910/DVN/4U4TTL.
